# Ultrasonographic Screening of Dairy Cows with Normal Uterine Involution or Developing Postpartum Uterine Disease Using B-Mode, Color, and Spectral Doppler

**DOI:** 10.1155/2023/2597332

**Published:** 2023-09-15

**Authors:** Mariana Henao-Gonzalez, María S. Ferrer, Claudia Jiménez-Escobar, Luis G. Palacio-Baena, Juan G. Maldonado-Estrada

**Affiliations:** ^1^One Health and Veterinary Innovative Research and Development (OHVRI-Group), College of Veterinary Medicine, University of Antioquia, Medellin 050034, Colombia; ^2^Department of Large Animal Medicine, College of Veterinary Medicine, The University of Georgia, Athens, Georgia, USA; ^3^Reproductive Biotechnology Laboratory, Section of Theriogenology and Herd Health, Department of Animal Health, Faculty of Veterinary Medicine and Zootechny, National University of Colombia, Bogota, DC, Colombia

## Abstract

Uterine involution, ovarian activity, and incidence of postpartum uterine disease (PUD) were assessed in forty-eight dairy cows from calving until the 10^th^ postpartum week. Postpartum follow-up included evaluation of uterine involution and ovarian structures by B-mode, Doppler color, and Doppler spectral ultrasound of the right uterine artery in cows with no calving or postpartum uterine problems (healthy cows). Data from cows that developed PUD (PUD cows) were compared with healthy cows matched by herd and days in milk (DIM). Data were analyzed by descriptive statistics, simple regression, one-way ANOVA, or repeated ANOVA measures, and in data analysis of healthy cows, uterine horn diameter assessed by B-mode ultrasound ranged from 22.9 ± 2.4 to 19.4 ± 1.4 mm and 19.9 ± 2.2 to 20.5 ± 2.3 mm from the fourth to the seventh postpartum week in the left and right uterine horns, respectively (*P* > 0.05). During the study, 15 and 7 cows had *corpus luteum* in the left and right ovaries, respectively. The mean time for the first postpartum CL was 30.1 ± 3.2 DIM (min 8, max 67 DIM). In data analysis of PUD cows, uterine blood flow assessed by color Doppler ranged from 7.4 ± 4.0 to 43.75 ± 10.3% in cows that developed PUD compared to 16.7 ± 11.0% in healthy cows (*P* > 0.05). No statistically significant changes were found in resistance index, pulsatility index, time-averaged maximum velocity, time-averaged mean velocity, or diastole/systole ratio (D/S) in cows that developed PUD compared to healthy cows (*P* > 0.05). Finally, no correlation was found between Doppler spectral parameters and uterine involution (*P* > 0.05). Our data suggest that cows receiving transition diets and exhibiting normal calving undergo a rapid macroscopic uterine involution and ovarian follicular dynamics resumption. Complete ultrasound evaluation provides valuable data for assessing uterine involution in postpartum dairy cows.

## 1. Introduction

Multiple variables affect the reproductive efficiency of dairy cows, one of the most critical being the negative energetic balance (NEB). NEB, among other things, is related to BCS, dry matter intake, metabolic status [1], heat stress [2], and food intake during the peripartum period [3]. The energy balance (EB) status affects reproductive performance, including first postpartum ovulation, postpartum uterine involution, and uterine immune response [4–7], resulting in impaired uterine involution [8]. The negative relationship between pronounced NEB and postpartum uterine disease (PUD) in dairy cows has been reported [9–11]. PUD includes clinical metritis (Met), puerperal metritis (PM), clinical endometritis (CE), subclinical endometritis (SE) [12, 13], and cervicitis [14, 15]. The lack of consensus about appropriate diagnostic tools for defining PUD [16, 17] results in a lack of accurate diagnosis and control strategies [18].

B-mode ultrasound exam allows a direct assessment of the postpartum uterus, improving Met and CE diagnosis [19]. Besides, successful evaluation of uterine circulation by color Doppler and spectral Doppler was reported [20]. Furthermore, uterine blood flow changes have been reported during the estrus cycle's follicular and luteal phases [21], superovulation, and early pregnancy [22, 23]. In addition, the blood flow of specific vessels can be semi-quantitatively evaluated through Doppler indices, which allow measurement of the resistance of peripheral vessels to blood flow (reviewed in [19]). In Doppler ultrasound values, S represents the maximum systolic flow and *M* represents the end-diastolic or minimum-diastolic flow. Doppler indices are calculated from two consecutive blood flow waves captured by the probe and set up according to the equipment software (see Figure 4 in [19]).

Krueger et al. [24] evaluated uterine blood flow and the pulsatility index (PI) of uterine arteries from calving to the twelfth postpartum week in dairy cows. Also, Heppelmann et al. [25] found changes in uterine artery diameter, time-averaged maximum velocity (TMAX), and PI within the first two postpartum weeks of Simmental cows. However, to our knowledge, no studies on Doppler parameters of uterine arteries in postpartum cows affected by PUD have been reported.

Our working hypothesis proposed that the uterine artery blood flow parameters of cows developing PUD differed from those of healthy postpartum cows. The objectives of this study were to evaluate uterine involution, ovarian structures, serum calcium, milk beta-hydroxybutyrate concentration, uterine and ovarian blood flow by B-mode ultrasound and color Doppler, and uterine artery Doppler spectral values in postpartum dairy cows that received transitional dietary management, had normal calving, and did not suffer from PUD. We evaluated uterine involution by ultrasonography and compared the color and spectral Doppler parameters between dairy cows that develop PUD or remain healthy between the first and the seventh postpartum week.

## 2. Materials and Methods

### 2.1. Approval

The University of Antioquia Institutional Board for Using Animal Subjects in Experimental Designs supplied ethical support for the project (approved by Act # 111, June 8, 2017). Also, all farmers signed informed consent.

### 2.2. Type of Study

We performed a descriptive analysis including forty-seven cows from four dairy farms of the northern region of Antioquia State (Colombia), including Donmatias, Entrerrios, and Santa Rosa de Osos municipalities, settled within 2400 and 2600 meters over sea level, and temperatures ranging from 6 to 16°C all along the year. Most cows were Holstein breed (*n* = 43, 91.4%), whereas the remaining 8.6% (*n* = 4) were Jersey or Holstein *x* Jersey crosses. We define sample size by convenience, comprising normal calving and clinically healthy cows with no prepartum diseases. Normal calving was defined as calving with no assistance from operators or veterinarians. At three weeks before the expected calving date, the cows received a transition diet consisting of 2 kg of concentrated commercial food containing 12% crude protein and 1.75 kcal of NEL (according to each farm: Preparto® Solla, Medellín, Colombia; or Preparto® Agrocolanta, Medellín, Colombia), with no mineralized salt supplementation. Postpartum cows calving from November 2018 to February 2019 were enrolled in the study and assessed for four months until completing the sample size, as mentioned earlier. Our study sampled cows during the dry season (November to February), characterized by slightly higher temperatures than in the rainy season. After calving, cows were fed in a rotational grazing system consisting of Kikuyu (*Cenchrus clandestinum*, former *Pennisetum clandestinum*) grass complemented with 1 kg of concentrated commercial food containing 16 to 18% crude protein and 1.75 kcal of net energy of lactation (NEL) (according to each farm: Solla-Leche-18%, Solla, Medellín, Colombia, or Fertileche-Selección-17%® Colanta, Medellín, Colombia) provided for each 4 to 5 L milk yield beyond 10 L milk yield. The variation in crude protein content is related to each farm's nutritional feeding management, which was adjusted individually according to forage availability, cows' body condition score, and milk yield. These dairies are not mix-ration-based systems [26, 27]. Mineralized salt supplementation (100–120 g/day) was based on 6 to 8% phosphorus content: 40% NaCl, 13.5% calcium, 6% phosphorus, 6% sulfur, 0.5% magnesium, 0.3% copper, 0.7% zinc, 0.015% iodine, 0.005% cobalt, 0.0075% selenium, and 0.06% fluoride, all on a minimal basis (Sollasal-6%®, Solla, Medellin, Colombia), or 26% NaCl, 12% calcium, and 8% phosphorus, 0.3% sulfur, 4% magnesium, 0.1% copper, 0.6% zinc, 0.009% iodine, 0.003% cobalt, 0.006% selenium, and 0.080% Fluor, all on a minimal basis (Fertisal-8® Colanta, Medellín, Colombia). Ad libitum water was provided.

At the beginning of the study, all cows preselected in each farm that exhibited normal calving were followed up for their postpartum outcome. All cows included in the study were within the first and seventh lactation. The calves' birth weight record was not considered for analysis because of the absence of dystocia.

### 2.3. Inclusion Criteria and Clinical Examination

**Figure 1:**
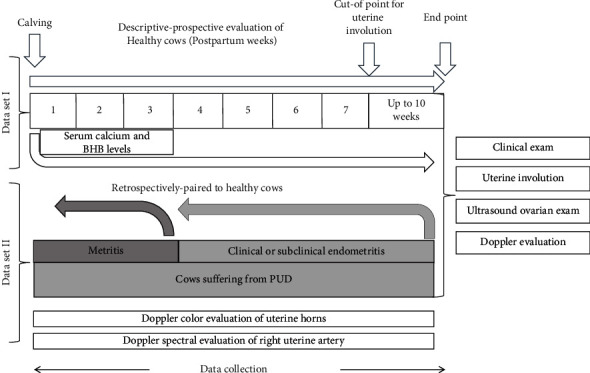
Study design and data collection schedule. All cows were evaluated from the first to the tenth postpartum week (descriptive study, dataset 1). Cows that developed PUD were matched to healthy cows for comparison purposes (retrospective analysis, dataset 2).

Each dairy farm was visited to select the prepartum cows included in the study. Once cows with normal calving and no clinical signs of metabolic diseases were selected, farms were visited from the first postpartum week and then weekly until the tenth postpartum week. At each visit, cows were evaluated by clinical exam and reproductive assessment by rectal palpation and ultrasound exam (Figure 1).

### 2.4. Clinical Exam

Cows were evaluated for their general condition to assess clinical signs indicative of metabolic or other prevalent diseases. Each cow was sampled at the time of inclusion and at each time point of evaluation, including blood and milk samples. According to the results at each time point of evaluation, cows were classified as healthy or developing PUD (Figure 1).

### 2.5. Clinical Reproductive Exam

Healthy cows had clinical parameters (heart rate, respiratory rate, body temperature, body condition score, mucosae color, appetite, and milk yield) within physiological conditions. They did not develop general disease or any PUD at each time point of evaluation. PUD case definitions were based on the reports in [28, 29]: (1) clinical metritis (Met), if a uterine infection did occur during the first twenty-two postpartum days, presenting uterine thickening and fetid purulent uterine content with vaginal discharge; (2) clinical endometritis (CE), if a uterine infection occurred after 23 days postpartum and the cow had mucopurulent to purulent material with or without vaginal discharge diagnosed by rectal palpation and ultrasonography; and (3) subclinical endometritis (SE), if a uterine infection was diagnosed after 40 days postpartum with no clinical signs during the reproductive exam; a cytology sample was obtained by cytobrush, which was smeared onto glass slides, fixed, and Wright-Giemsa stained, and uterine cells and polymorphonuclear neutrophils (PMN) were counted under a microscope [30, 31]. The cutoff point was the presence of more than 5% PMNs in a 200-cell count. No information about uterine cervicitis was recorded in the study (Figure 1).

### 2.6. Blood and Milk Sampling

Blood samples were taken from the coccygeal vein using a vacutainer with no anticoagulant. Blood samples were transported to the laboratory under ice and centrifuged to obtain serum. Serum was stored at −4°C until processing. Calcium concentration was measured using an automated serum analyzer (Mindray autoanalyzer IN-B2400 Plus) with Randox calcium kits (Randox Laboratories Canada Ltd.) in a certified diagnostic laboratory in Rionegro (Antioquia, Colombia). Milk samples were assessed for BHB (mM) using infrared spectroscopy (MilkoScan FT2, Foss Analytics) at the Raw Milk Quality and Safety Laboratory of the University of Antioquia (Medellin, Colombia).

## 3. Postpartum Uterine Involution and Ovarian Status

### 3.1. Uterine Involution

**Figure 2:**
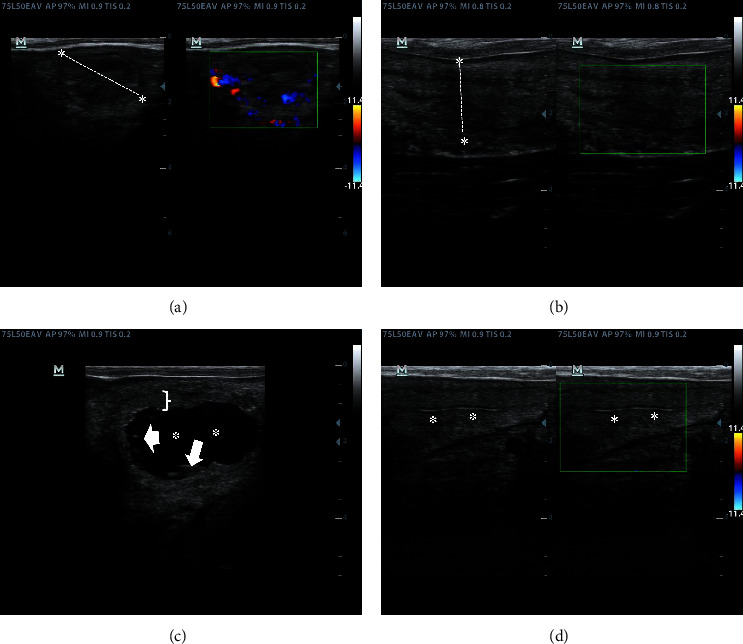
B-mode and Doppler color ultrasound evaluation. (a, b) Cross section and longitudinal measurements of uterine diameter (a dashed line between asterisks) in the uterine horn and its corresponding Doppler color image. (c) B-mode ultrasound image of a uterine lumen having a nonechogenic fluid (asterisks) and floating debris (arrows) corresponding to caruncular involution. The uterine mucosa is shown in the bracket. (d) B-mode and color Doppler ultrasound of the uterine horn in a longitudinal section showing intraluminal hyperechogenic material (asterisks).

Cows meeting eligible criteria and selected for the study started at 3 ± 2 days postpartum were evaluated by ultrasound using a Mindray Z5 portable ultrasound equipped with a 5.0 to 7.5 MHz gradable probe (Mindray Medical Colombia, Marquetingnet S.A.S., Bogotá, Colombia). Each cow was assessed by a transrectal exam, including an exhaustive evaluation of the uterine horns with cross-sectional and longitudinal images captured by B-mode ultrasound. In addition, cross section uterine horn images taken at the greater uterine curvature were recorded. The cursor was set up at the uterine curvature to measure the distance between the dorsal, ventral, and lateral spaces corresponding to the uterine mucosa's 3 and 9-o'clock sides (Figure 2). The same veterinarian (MHG) performed all measurements. The average measurements were considered as the diameter of each uterine horn. Also, the presence of uterine content indicated by a hyperechogenic line or variable amounts of hyperechogenic content was deemed abnormal and indicative of CE if corroborated by positive endometrial cytology.

According to the clinical progress of the reproductive tract of the cows, two datasets were obtained. Dataset one depicts findings of healthy cows that remained nonaffected by general disease or PUD through the study period. Tables 1 and 2 show overall (*n* = 27) and specific farm data, respectively. Dataset 2 depicts findings of cows with normal calving that developed Met, CE, or SE during the follow-up period (*n* = 15). See Tables 1 and 2 for overall and specific farm data, respectively. In addition, PUD cows were retrospectively matched to healthy cows by DIM and farm for data comparison purposes (Figure 1).

### 3.2. Ovarian Follicles and Corpus Luteum Assessment

**Figure 3:**
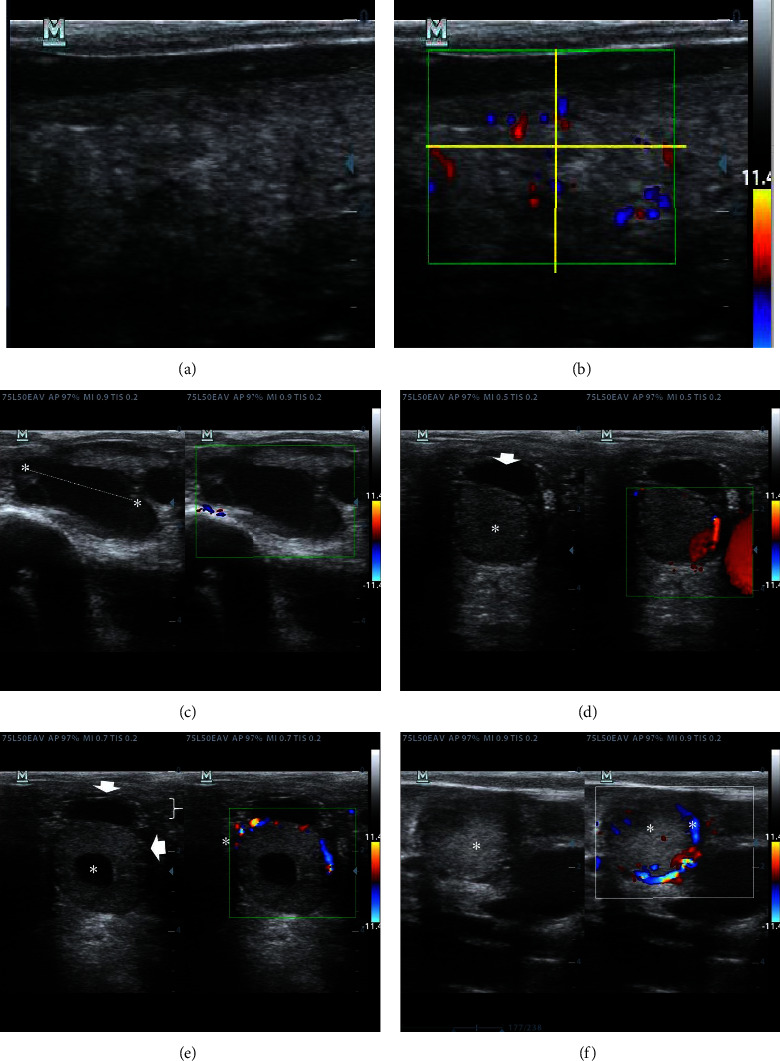
B-mode (a) and color Doppler (b) ultrasound images of the uterine horn. A box (green line) was drawn over the image of the uterine horn in B-mode ultrasound, and a cross (yellow line) was drawn to define the four quadrants in which blood flow was evaluated by color Doppler mode (b). B-mode and color Doppler ultrasound image of a large follicle and a weak blood flow signal is shown in (c). B-mode and color Doppler ultrasound image of a corpus luteum with appreciable blood flow signal is shown in (d) with a medium-sized follicle over the corpus luteum (white arrow). (e) and (f) represent the B-mode and Doppler color pattern of a cavitary (asterisk) and a normal (asterisk) corpus luteum, exhibiting their corresponding weak blood flow.

The ovaries were analyzed for the presence of follicles and corpus luteum by rectal palpation followed by B-mode, color Doppler, and spectral Doppler ultrasound. Findings were recorded for each cow. Only the greatest follicle was measured in each ovary, and any corpus luteum detected was also recorded, although the corpus luteum diameter was not recorded (Figure 3).

### 3.3. Doppler Color Assessment of Uterine Horns, the Greatest Dominant Follicle, and Corpus Luteum

**Figure 4:**
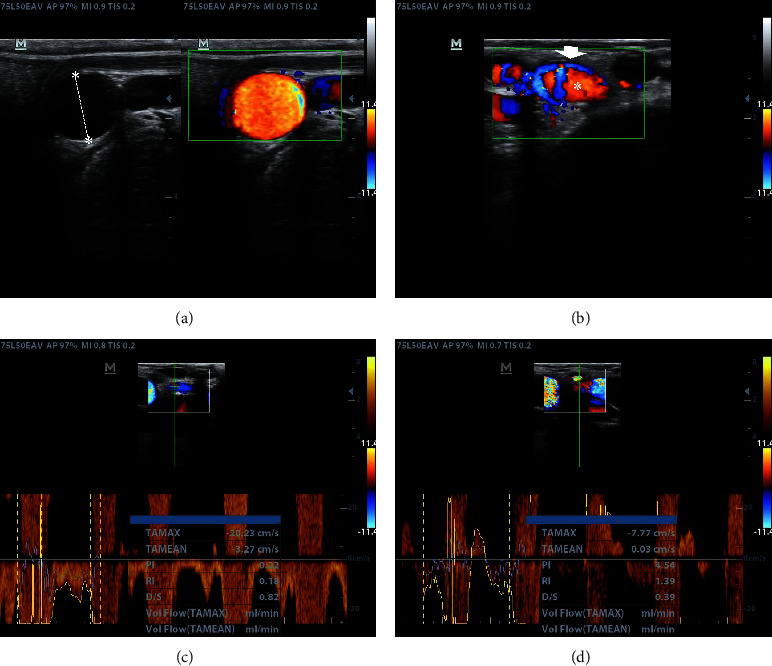
The sequence of uterine artery localization by B-mode and Doppler color of a 35 DIM dairy cow. (a) Internal iliac artery next to the umbilical artery. (b) Doppler color assessment of the above structures (dashed green box). (c, d) Doppler spectral assessment of the right uterine artery and datasets provided by the ultrasound machine (IR, IP, TAMAX, TMEAN, and D/S).

To collect the color Doppler data, a box (green line in Figures 2–4) was drawn over the image of the uterine horn in B-mode ultrasound. Next, a cross (yellow lines in Figure 3) was drawn to define the four quadrants where blood flow was evaluated in color Doppler mode. Blood flow was then recorded in the resulting quadrants. Next, the data were taken in pixels and transformed into flow percentages in each quadrant by the equipment software. Finally, the final flow values for each quadrant were averaged, and the values were evaluated as mean ± standard error of the mean.

### 3.4. Doppler Spectral Assessment of the Uterine Artery

The equipment was adjusted for Doppler spectral parameters to evaluate the uterine arteries. The right uterine artery was assessed in each cow within a 5-minute average duration. The lineal transductor was put in a dorsal-oriented cross-sectional orientation on the uterine artery, according to Bollwein et al. [19]. In brief, the aorta artery was found with the probe, which was moved caudally until the origin of the external iliac artery, which follows ventrally aside from the iliopectineal line. Then, the internal iliac artery was set caudally (Figure 4), followed by the common trunk, before branching the umbilical and uterine arteries. Otherwise, the middle uterine artery is palpated as a mobile vessel in the mesometrium. Following its origin in the umbilical artery, we visualized and evaluated the uterine artery. Doppler spectral parameters were recorded with the US probe at a 60% angle for more accurate measurement [19, 32]. Doppler parameters recorded were resistance index (RI), pulsatility index (PI), time-averaged maximum velocity (TMAX), time-averaged mean velocity (TMEAN), and systole-to-diastole ratio (S/D) (Figure 4).

### 3.5. Endometrial Cytology

Endometrial samples were taken by cytobrush, smeared on glass slides, and stained by Wright staining. The percentage of PMN after counting two hundred cells in 40x magnification was calculated as previously described, considering positive values greater than 5% PMN [30].

### 3.6. Statistical Analysis

For data analysis, data from forty-three cows in which three or four consecutive evaluations were performed were finally included for analysis. Data from Doppler measurements—RI, PI, TMAX, TMEAN, and S/D, the left and right uterine horn diameter, and serum calcium concentration were assessed for normal distribution and homogeneity of variance using Levine's test. These variables fit a normal distribution. Comparisons of Doppler values according to ovarian findings, e.g., between cows exhibiting *corpus luteum*, only follicles, or no ovarian structures, were compared by one-way ANOVA, including Tukey HSD. A corpus luteum presence was compared between cows suffering PUD and healthy paired cows by chi-square test with Yates's correction. Comparison of nonparametric data from color Doppler quadrants of the left and right ovaries and uterine horns between cows suffering PUD and healthy paired cows were analyzed by *T*-Test Calculator for two independent means. It was also applied to compare uterine horn diameter and Doppler spectral values between cows exhibiting corpus luteum, follicles, or no structures to assess if ovarian structures had a statistically significant effect on these variables. One-way ANOVA analyzed the comparison between PUD incidences farm by farm. Finally, Doppler parameter values of healthy cows between postpartum weeks 1 and 7 were evaluated by repeated ANOVA measures. This analysis did not apply to data from PUD cows due to few case numbers (Table 2).

## 4. Results

The mean body condition score (BCS) of cows included in the analysis was 3.0 (1.0 to 5.0 scale) (data not shown). The average parity of healthy cows was 2.65 (mean ± SEM) (Table 1). Twenty-seven cows (62.79%) did not develop PUD and were in the group of healthy postpartum cows. In contrast, sixteen episodes of PUD were found (37.2%), including clinical metritis (*n* = 4), clinical endometritis (*n* = 7), and subclinical endometritis (*n* = 5) (Tables 1 and 2). No cases of puerperal metritis were diagnosed. No statistically significant differences were found when comparing parity and DIM data from healthy cows to cows that developed PUD according to farm (Table 2). Interestingly, no cases of PUD were found on farm one. Due to sample size, no statistically significant differences were assessed for PUD between farms (Table 2).

### 4.1. Dataset One: Uterine Involution in Cows Presenting Normal Calving with No PUD

Data were grouped into weekly intervals for comparison even though data were recorded since the first postpartum week; only since the fourth week postpartum were we able to record uterine diameter data from healthy cows not developing PUD (Table 3).

### 4.2. Serum Calcium and Milk Beta-Hydroxybutyrate (Beta-OHB) Concentration

**Figure 5:**
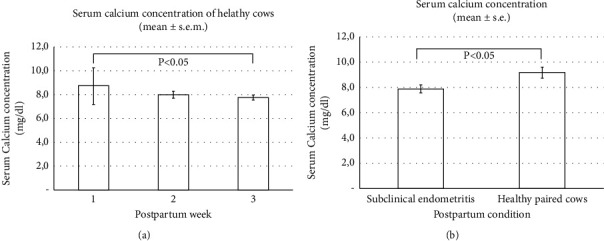
Serum calcium concentration in postpartum weeks 1 to 3 in dairy cows with normal calving that have not developed the postpartum uterine disease, PUD (a), or cows that developed PUD (b). Values are expressed as mean ± SEM.

Average calcium values were 8.2 ± 0.8 mg/dl (*n* = 24), with two cows (4%) presenting hypocalcemia values. Serum calcium concentration ranged from 8.7 ± 1.5 to 7.8 ± 0.2 (mg/dl) from the first to the third postpartum week, with no statistically significant differences (*P* > 0.05) between weeks (Figure 5(a)). The average beta-OHB values were 0.05 (±0.02 SEM). According to clinical findings and hyperketonemia values, we found no cows with clinical or subclinical ketosis (data not shown).

### 4.3. Assessment of Uterine Involution

The diameter of the right (RUH) and left (LUH) uterine horns recorded from the fourth to the seventh postpartum weeks did not significantly differ during the study period nor between the right and the left horns (*P* > 0.05). The uterine horn diameter ranged from 19.4 to 22.9 mm (Table 3). These values remained without statistically significant differences up to the study's endpoint at the seventh postpartum week (Supplementary Figures 1A and 1B). No cases of delayed uterine involution were found in these healthy postpartum cows.

### 4.4. Ovarian Follicles and First Postpartum Ovulation

Ovarian follicles were first detected in the second and third postpartum weeks onward. The average (± standard deviation) follicle diameter was 10.6 ± 5.9 and 11.8 ± 4.7 for the right and left ovaries, respectively. Two cows presented cystic follicles (diameter greater than 30 mm). Besides, we found an early resumption of ovarian activity (36.67% of the cows), evidenced by the presence of dominant follicles (follicles with >8 mm) as early as the third week postpartum. The average age for corpus luteum presence was 30.1 ± 3.2 DIM (± standard error) (minimum 8, maximum 49 DIM) in 13 out of 41 healthy cows with no PUD. In twenty-two out of 60-time point measurements (36.6%), a *corpus luteum* was detected. There were seven cows with CL in the right ovary at 50.3 ± 5.4 DIM (min 22, max 65 DIM) and 15 with CL in the left ovary at 43.7 ± 4.5 DIM (min 8, max 70 DIM). The first postpartum corpus luteum (CL) was found as early as 8 DIM in a cow.

### 4.5. Doppler Color Findings

We do not regularly detect blood flow in the ovaries and uterine horn quadrants (Table 4). Most cows presented blood flow between the fourth and sixth postpartum weeks with no statistically significant differences between the right and left uterine horns (*P* > 0.05) and the right and left ovaries (*P* > 0.05). No statistically significant differences were found when comparing data between postpartum weeks (*P* > 0.05). Most of the cows presented higher values of Doppler color in CL quadrants than in follicle quadrants (Figures 3(d)–3(f)). Blood flow statistical analysis could not be performed for the largest follicles because most cows with follicles in their ovaries exhibited no Doppler color signals (data not shown).

### 4.6. Doppler Spectral Parameters

#### 4.6.1. Resistance Index (RI)

RI values showed a high variability during the evaluation period (Supplementary Figure 2). RI values ranged from 0.98 to 0.9 between the first and seventh weeks postpartum (Table 3), with no statistically significant differences (*P* > 0.05). RI values showed a slight increase from the first to the second postpartum week, followed by a decline up to the fifth, increased until the eighth week, and then declined again in the ninth week, with a final increase up to the tenth postpartum week (Supplementary Figure 2A), fitting a six-degree polynomial trend (*y* = 0.0001 × 6 − 0.0037 × 5 + 0.0323 × 4 − 0.0922 × 3 − 0.0598 × 2+ 0.4186x + 0.691; *R*^2^ = 0.855).

#### 4.6.2. Pulsatility Index (PI)

PI values ranged from 2.7 to 8.3 between the first and seventh weeks postpartum (Table 3), with no statistically significant differences (*P* > 0.05). PI decreased from the second to the fourth postpartum week, increased from the fourth to the seventh, and then declined in the ninth and tenth postpartum weeks (Supplementary Figure 2B), fitting a six-degree polynomial trend (*y* = 0.0018 × 6 − 0.0484 × 5 + 0.4509 × 4 − 1.7084 × 3 + 2.051 × 2 + 0.9396*x* + 0.921; *R*^2^ = 0.6556).

#### 4.6.3. TMAX

TMAX values (ml/sec) ranged from 0.75 to −0.7 between the first and seventh postpartum weeks (Table 3), with no statistically significant differences (*P* > 0.05). TMAX decreased from the first to the fifth postpartum week, increased from the fifth to the eighth week, and declined in the tenth postpartum week (Supplementary Figure 3B), fitting a six-degree polynomial trend (*y* = 0.0066 × 6 − 0.2051 × 5 + 2.4651 × 4 − 14.37 × 3 + 42.161 × 2 − 59.264x + 29.854; *R*^2^ = 0,8881).

#### 4.6.4. TMEAN

TMEAN values (ml/sec) ranged from 3.59 to 1.6 between the first and seventh postpartum weeks (Table 3), with no statistically significant differences (*P* > 0.05). Similarly, TMEAN decreased from the first to the fifth postpartum week, increased from the fifth to the eighth, declined in the ninth week, and increased in the tenth postpartum week (Supplementary Figure 3B), fitting a six-degree polynomial trend (*y* = 0.0035 × 6 − 0.1139 × 5 + 1.4291 × 4 − 8.8709 × 3 + 28.503 × 2 − 44.962x + 27.553; *R*^2^ = 0.9185).

#### 4.6.5. D/S

D/S values ranged from 0.93 to 0.7 between the first and seventh postpartum weeks (Table 3), with no statistically significant differences (*P* > 0.05). D/S values showed a weekly oscillatory pattern from the first to the tenth postpartum weeks (Supplementary Figure 4A), fitting a six-degree polynomial trend (*y* = 0.0007 × 6 − 0.0228 × 5 + 0.3081 × 4 − 2.0567 × 3 + 7.0175 × 2 − 11.244x + 6.9225; *R*^2^ = 0.6865).

### 4.7. Dataset Two: Postpartum Uterine and Ovarian Parameters in Cows That Developed PUD Compared to Healthy Cows

In this study, the postpartum day at which normal calving cows presented any episode of PUD was recorded, and data from their uterine and ovarian follow-up were retrospectively compared to data from normal calving cows with a healthy postpartum condition. Data from cows that develop PUD were matched to data from healthy cows by DIM and farm. Four, seven, and five cows developed Met, CE, and SE (Table 1), with no statistically significant differences for DIM (*P* > 0.05) when compared to healthy postpartum cows. However, serum calcium concentration was significantly lower (*P* < 0.05) in the dataset of cows that developed SE (7.88 ± 0.3 mg/dL) compared to the dataset from healthy postpartum cows (9.17 ± 0.4 mg/dL) (Figure 5(b)). Besides, several cases of PUD occurred in the same cows during the follow-up period: three cows developed Met and then CE and two cows presented two or three episodes of SE.

The diameter of the right uterine horn (Supplementary Figure 1C) and left uterine horn (Supplementary Figure 1D) did not significantly differ from the corresponding values of healthy postpartum cows (*P* > 0.05). The ultrasonography exam shows that all cows evaluated presented a complete macroscopic uterine involution at the fourth postpartum week. Uterine horn diameters ranged around 23.57 ± 3.8 and 18.10 ± 1.3 mm and 19.4 ± 2.4 and 17.25 ± 1.4 mm for the right and the left uterine horns in cows that developed Met and CE, respectively.

#### 4.7.1. Ovarian Findings

In cows that developed Met, CE, and SE, the follicle diameter ranged from 3.8 to 15 mm, 7.8 to 21.2 mm, and 9.8 to 15.7 mm, respectively. In cows with normal calving that developed PUD, we found the first CL at 25.8 ± 6.2 (min 9, max 35 DIM). One of the cows that developed Met had corpus luteum, whereas two and three cows that developed CE and SE showed corpus luteum. In addition, one healthy cow and two healthy cows matched to CE and SE, respectively, showed corpus luteum (no statistical analysis could be performed).

#### 4.7.2. Doppler Color Ultrasound

Color Doppler values of the right and left uterine horns were 55.5 ± 20.8 (*n* = 9) and 50 ± 20.4 (*n* = 7) respectively, in cows that developed PUD, compared to 35.7 ± 19.6 (*n* = 7) and 46.4 ± 22.49 (*n* = 7) respectively, in healthy cows, with no significant statistical differences (*p* > 0.05).

#### 4.7.3. Doppler Spectral Ultrasound

RI (Supplementary Figure 2A), PI (Supplementary Figure 2B), TMAX (Supplementary Figure 3A), TMEAN (Supplementary Figure 3B), and D/S (Supplementary Figure 4B) values did not significantly differ between cows that developed PUD and healthy cows (*P* > 0.05). Doppler spectral values were compared between farms and ovarian status, including ovaries with no structures, only follicles, and ovaries with corpus luteum. No statistically significant differences were found (data not shown). Only RI values were significantly higher in cows having a corpus luteum (*P* < 0.05) compared to cows with no structures or only follicles in their ovaries (Supplementary Figure 5).

## 5. Discussion

This study evaluated uterine involution in dairy cows presenting normal calving in high-altitude tropical dairies using B-mode, Doppler color, and spectral ultrasound. Besides, in a retrospective analysis, we evaluated uterine involution in a group of cows that developed PUD. We compared it with data from healthy postpartum cows matched to PUD by farm and DIM. All cows enrolled in the study showed complete macroscopic uterine involution at the fourth week postpartum based on uterine horn diameter assessed by B-mode ultrasound. Serum calcium concentration did not significantly differ in healthy postpartum cows during the three postpartum weeks. Still, cows with SE but not Met or CE had significantly lower calcium concentrations than their matched healthy cows (*P* < 0.05).

In this study, cows that developed Met or CE showed rapid macroscopic uterine involution, as evidenced by uterine horn diameters in both uterine horns from the fourth week postpartum, as reported previously [33]. This finding could result from the objective inclusion criteria for the study, highlighting the importance of providing the cows with a transition diet for improving postpartum immune response and metabolic status [34, 35]. Besides, PUD is a risk factor for culling lactating dairy cows due to its negative impact on pregnancy rates [36–38]. Interestingly, we performed a B-mode ultrasound evaluation of uterine involution, which provided a confident result for the absence of abnormal uterine contents and accurate measurements of the uterine horn's diameter. Furthermore, even though cows that developed Met were diagnosed on average at 12 days postpartum, their macroscopic uterine involution was not significantly affected compared to healthy matched cows (Supplementary Figure 1) in agreement with Kawashima et al. [8].

The curve for uterine diameter presented a polynomial trend for the left and right uterine horns (data not shown). This finding agrees with Okano and Tomizuka [39], who found a polynomial trend in uterine horn diameter, uterine body, and cervix diameter in normal calving Holstein dairy cows evaluated from the third to the 48^th^ DIM. These authors reported uterine diameters around 3.2 cm in the previously pregnant and nonpregnant uterine horns at 21^st^ DIM. Our study's diameter was around 2.2 cm on the same postpartum days (Supplementary Figure 1). Our results on uterine horn diameter agree with the report by Scully et al. [40], who evaluated uterine involution in lactating and nonlactating primiparous Holstein dairy cows, reporting 25 mm diameter at 21 postpartum days in the previously gravid uterine horn [40], and findings correspond to cows having normal calving as performed in our study (Figures 2(a) and 2(b)).

Cows were evaluated from the first postpartum week onward, and we were primarily focused on uterine involution, although data collection from ovaries could be achieved from a few cows. However, recording data from all cows was possible since the first postpartum day. In addition to great uterine content in cows with Met, we also found a hyperechogenic content in the uterine lumen in cows that showed no signs of CE at first, a finding further accompanied by positive endometrial cytology, suggesting that some cows that developed CE did not necessarily exhibit purulent vaginal discharge (PVD). This finding highlights the importance of using ultrasound in experimental studies on postpartum uterine diseases. As much as possible, its use must be encouraged to be progressively incorporated as a diagnostic tool by the bovine practitioner.

In our study, 35% of cows presented subclinical hypocalcemia with no cases of milk fever. Three studies reported a relationship between subclinical hypocalcemia and Met [41, 42]. Only 10% of cows in our study developed Met due to the inclusion criteria that granted normal calving cows entering the study. However, our research did not evaluate a relationship between low calcium concentration and Met.

We found an early resumption of ovarian follicular function in both ovaries according to the presence of dominant follicles and early postpartum ovulation in at least a third of cows.

The indices included in the present study comprise the time-averaged peak velocity (TAMEAN or TMEAN), the time-averaged maximum velocity (TMAX), the pulsatility index (PI), the resistance index (RI), and the S/D ratio [43, 44]. PI and RI represent the semi-quantitative assessment of blood flow resistance. While S/D and RI show a parabolic relationship with increased vascular resistance, PI shows a linear correlation with vascular resistance [19]. Resistance refers to factors that impede or reduce blood flow, e.g., resistance faced by blood flow as it passes through a blood vessel. High resistance flows are more pulsatile and correspond to extremities arteries and sites not requiring constant blood flow, exhibiting low diastolic values and a reverse flow area in diastole. Therefore, this fact could apply to the uterine artery during uterine involution in the postpartum dairy cow.

Doppler spectral parameters found in healthy postpartum cows were like Herzog and Bollwein's report [45], which found reduced blood flow during the first postpartum week in healthy cows. Uterine blood flow appears to vary in response to phases of the estrus cycle and the reproductive status of the cow, probably because of the predominant hormonal milieu in the uterus, with a positive relationship found between progesterone concentration and RI values [46], and it was found to be related to heat stress [47, 48] but not in our study. RI and PI are the most common parameters evaluated for studying uterine blood flow. If RI values are low, it shows increased uterine blood flow [19]. Nevertheless, we found no statistically significant differences between cows suffering PUD and healthy postpartum cows.

Kaya et al. [49] found no correlation between Doppler parameters, follicular dynamics, and ovulation in cows exhibiting a PGF2a-induced estrus. Contrary to the finding by Moonmanee et al. [50], who reported a high relationship between ovarian blood flow dynamics and reproductive parameters in beef cows, even though we did not evaluate the progesterone or estrogen concentration in our study, we found that cows with *corpus luteum* showed significantly higher values of RI (*P* < 0.05) than cows with no ovarian structures or follicles. This parameter is also affected by gestational age and hormonal status [51, 52], environmental temperatures [53], and other not evaluated factors in the present study. The RI found in healthy cows presents an oscillatory pattern of three weeks, representing blood flow fluctuations related to the increased ovarian follicular activity and the elevation of circulating estrogens. Estrogen receptors in the tunica media of uterine arteries respond to calcium concentration [54], influencing vessel contractibility and blood flow. In the report by Debertolis et al. [20], the authors reported a negative relationship between TMAX and PI, with increased TMAX, indicating a high blood flux in cows with acute endometritis. The authors also found that TMAX significantly decreased in the fourth and fifth postpartum weeks and then increased in the seventh week, agreeing with our TMAX results (Supplementary Figure 3A), which reached a lower value in the fifth and sixth postpartum week and then increased in the seventh postpartum week.

Interestingly, TMEAN values showed a similar pattern (Supplementary Figure 3B), representing the uterine involution period until ovarian follicular dynamics resumption. Besides, our study's average DIM at the first postpartum corpus luteum was 39.6 ± 4.6 days. Progesterone and estrogen serum concentrations play a critical role in uterine blood flow by their effect on uterine arteries; estrogens cause vasoconstriction of the arterial smooth muscle, and progesterone causes vasodilation [19, 20]. Although no hormonal measurements were performed in this study to evaluate if the presence of ovarian structures could affect Doppler spectral parameters (as indirect evidence of the steroid hormonal status), we found no statistically significant differences in Doppler spectral data between cows with no ovarian structures, cows with dominant follicles, or cows with *corpus luteum* (data not shown). These data support the concept that the ovarian status of the cows did not significantly affect Doppler spectral parameters in our sample. Only the resistance index was significantly increased (*P* < 0.05) in healthy cows, showing *corpus luteum* compared to cows with no ovarian structures and cows having only follicles (Supplementary Figure 5). No comparisons could be evaluated with cows suffering PUD due to the low sample size in these groups.

Blood flux volume was significantly reduced proportionally to DIM due to progressive uterine size and weight reduction resulting from uterine involution. In contrast, IP values decreased to 30 DIM due to caruncular vascular supply reduction. In this context, PUD may alter the pattern of uterine blood flow [19, 25]. According to our results, cows reached a complete macroscopic uterine involution within the fourth postpartum week. However, values of RI increased between the fifth and seventh postpartum week, a finding that could reflect decreased blood flow, probably because several cows exhibited a *corpus luteum* early postpartum. RI increased due to a high circulating progesterone concentration and reduced blood flow. On the contrary, when luteolysis occurs, RI values decrease with progesterone concentration. Accordingly, Hassan et al. [46] reported a proportional relationship between RI and progesterone.

In cows with CE, the Doppler parameters were more variable, where the PI was higher in CE cows than in healthy matched cows, while the RI was similar in both groups (*P* > 0.05). The results on blood flow are like other studies where authors found increased PI and RI in severe and moderate cases of CE. In this study, the higher PI value in CE cows compared to healthy cows suggests PI could be increased in cases of CE, in agreement with the report by Sharma et al. [55] and with the finding of Doppler color in CE.

The present study provides evidence of rapid uterine involution in cows with normal calving, some presenting a single or several repeated Met and CE episodes. No statistically significant differences were found for the average diameter of both uterine horns, ranging from 25 to 20 mm from the fourth to the seventh postpartum week. This suggests a rapid macroscopic involution almost completed after the fourth postpartum week. No differences in the diameter of uterine horns were found between cows that developed PUD compared to matched healthy cows. At least at the beginning of the third postpartum week, we found dominant follicles in both ovaries, accompanied by rapid detection of the first postpartum *corpus luteum*, in 36.9% of cows not developing PUD on average at 30 postpartum days, compared to 16% of cows suffering Met or CE on average at 46 postpartum days. An intriguing result was the finding of rapid macroscopic involution of uterine horns in cows that developed PUD. Further studies must include comparing uterine ultrasound parameters in cows with normal calving or dystocia and comparing the frequency of PUD according to calving status.

In conclusion, this study provides evidence of evaluating postpartum uterine involution by B-mode ultrasound evaluation of uterine horns and Doppler ultrasound and Doppler spectral of the uterine artery. Our results evidenced a rapid involution of the uterus in cows calved with no complications, where almost 100% of cows presented a complete macroscopic uterine involution at the fourth postpartum week. Also, Postpartum involution finished earlier than one month even in cows presenting PUD, a finding suggesting the time elapsed between the diagnosis of PUD and its remission was lower than one week. One concluding remark of our study is using ultrasonography for a more accurate diagnosis of uterine involution, ovarian follicular dynamics and ovulation, and PUD diagnosis.

## 6. Strengths and Weaknesses of the Study

One strength of the study is the assessment of uterine involution using an objective measurement with B-mode ultrasound, which calls for an exact diagnosis. Similarly, ultrasound allowed us to detect the cases of CE otherwise not seen because there was not enough endometrial content to be observed by clinical inspection. In addition, finding early postpartum *corpus luteum* in some cows allows for a search for those cows that could be programmed for insemination early in the voluntary waiting period, once the microscopic involution is reached, from 40 to 45 days onward. This finding resembles cases where cows that show second estrus before 60 days postpartum receive a fertile insemination whose successful pregnancy results in shorter calving intervals. This fact must encourage increasing B-mode ultrasound examinations to assess dairy cattle's postpartum uterine diagnosis.

This study performed no hormone measurements to corroborate the reestablishment of follicular dynamics, which were only evaluated by ultrasound examination. Hormonal measures could provide evidence of interactions between hormones and Doppler spectral values as reported for cycling dairy cows. However, because Doppler spectral and Doppler color findings were inconclusive, these measurements must be evaluated in a cohort including cows with dystocia compared to cows with normal calving.

Further studies should evaluate the behavior of uterine involution variables and ultrasonographic evaluation in cows that have not received the transition period diet and compare the clinical outcome at calving and during the first 45 days postpartum to determine if there are differences between cows that present calving problems such as dystocia and retained placenta and cows that do not offer these conditions. In addition, further studies will allow us to evaluate whether there is a relationship between the degree of loss of postpartum body condition score and the incidence of metabolic problems and their impact on ultrasonographic parameters such as those evaluated in this study.

## Figures and Tables

**Table 1:** The study included epidemiological data of cows with normal calving and healthy postpartum from high-altitude tropical dairy herds.

Group	Number of cows	Parity			DIM		
	*n*	Mean ± SEM	Min	Max	Mean ± SEM	Min	Max
Healthy cows	26	2.65 ± 0.4	1	7	36.9 ± 17.9	2	70
Cows suffering Met	4	5.25 ± 0.9	3	7	12.0 ± 2.5	7	18
Healthy matched to Met^*∗*^	3	2.0 ± 0.6	1	3	12.3 ± 3.9	7	20
Cows suffering CE	7	3.9 ± 0.7	1	6	35.7 ± 6.3	24	69
Healthy matched to CE^*∗*^	7	1.9 ± 0.3	1	3	34.4 ± 4.6	24	55
Cows suffering SE	5	1.4 ± 0.2	1	2	52.2 ± 7.8	35	71
Healthy matched to SE^*∗*^	5	3.4 ± 0.9	2	6	50.4 ± 6.9	35	68

^*∗*^Cows affected by Met, CE, or SE were matched by farm and DIM to healthy cows (*P* > 0.05). DIM, days in milk. Met, metritis. CE, clinical endometritis. SE, subclinical endometritis.

**Table 2:** Days in milk and parity values of cows of the study comparing healthy cows with cows developing PUD.

Healthy cows					Cows developing PUD			
Farm	Cows preselected	Cows evaluated	DIM at evaluation, mean ± SEM (min, max)	Parity, mean ± SEM (min, max)	Cows evaluated	DIM at evaluation, mean ± SEM (min, max)	Parity, mean ± SEM (min, max)	*P*
1	10	7	38.9 ± 6.6 (5, 70)	2.3 ± 0.3 (1, 3)	0	—	—	n.a.
2	7	4	41.5 ± 1.4 (20, 64)	2.5 ± 0.5 (2, 3)	5	26.8 ± 6.4	3.8 ± 0.5 (3, 5)	*P* > 0.05
3	7	5	35.9 ± 3.2 (2, 63)	2.3 ± 0.3 (1, 5)	3	52.3 ± 10.4 (35, 71)	1 (1, 1)	*P* > 0.05
4	22	10	38.9 ± 6.6 (6, 54)	2.5 ± 0.9 (1, 7)	7	35.5 ± 8.3 (7, 69)	3.9 ± 0.9 (1, 7)	*P* > 0.05
Total	46	26	38.0 ± 14.0 (2, 70)	2.5 ± 0.9 (1, 7)	15	34.9 ± 6.4 (7, 71)	3.3 ± 0.5 (1, 7)	*P* > 0.05

DIM: days in milk. SEM: standard error of the median. PUD: postpartum uterine disease. n.a.: not assessed.

**Table 3:** Ultrasound and Doppler spectral values of cows with normal calving and healthy postpartum from high-altitude tropical dairy herds.

Parameter	Postpartum weeks^*∗*^													
	*n*	1	*n*	2	*n*	3	*n*	4	*n*	5	*n*	6	*n*	7
DIM (days)	3	4.3 ± 1.2	4	9.3 ± 0.8	6	18.7 ± 0.6	7	24.9 ± 1.0	7	33.1 ± 0.9	7	39.7 ± 0.9	7	45.3 ± 0.9
LUH (mm)		n.r.		n.r.		n.r.	6	22.9 ± 2.4	7	19.5 ± 1.9	7	20.6 ± 1.2	7	19.4 ± 1.4
RUH (mm)		n.r.		n.r.		n.r.	6	19.9 ± 2.2	7	22.4 ± 1.5	7	21.3 ± 1.4	7	20.5 ± 2.3
RI	5	0.98 ± 0.3	4	1.01 ± 0.2	6	0.63 ± 0.2	7	0.7 ± 0.2	7	0.5 ± 0.2	7	0.8 ± 0.1	7	0.9 ± 0.2
PI	5	2.7 ± 1.22	4	2.7 ± 0.14	6	2.65 ± 1.12	7	2.0 ± 0.9	7	1.3 ± 0.8	7	4.4 ± 1.7	7	8.3 ± 6.7
TMAX	5	0.75 ± 4.4	4	−2.17 ± 4.1	6	−1.18 ± 4.6	7	−4.1 ± 4.0	7	−7.2 ± 5.0	7	−4.7 ± 2.8	7	−0.7 ± 4.8
TMEAN	5	3.59 ± 1.75	4	−0.13 ± 1.89	6	0.75 ± 1.39	7	−0.4 ± 1.7	7	−1.2 ± 1.8	7	−0.4 ± 1.3	7	1.6 ± 2.1
D/S	5	0.93 ± 0.4	4	0.28 ± 0.13	6	0.77 ± 0.22	7	0.6 ± 0.2	7	0.8 ± 0.2	7	0.3 ± 0.2	7	0.7 ± 0.3
Ca^2+^ (mg/dL)	2	8.7 ± 1.5	4	8.0 ± 0.3	5	7.8 ± 0.2		—		—		—		—

DIM: days in milk. RUH: right uterine horn diameter. LUH: left uterine horn diameter. FRO: follicle diameter in right ovary. FLO: follicle diameter in left ovary. RI: resistance index. PI: pulsatility index. TMAX: maximal blood flow. TMEAN: mean blood flow. D/S: diastole-to-systole ratio. Values are expressed as mean ± SEM. ^*∗*^There were no statistically significant differences within weeks (*P* > 0.05). n.r.: not recorded.

**Table 4:** Mean percentage of blood flow in four quadrants of Doppler color ultrasound images from uterine horns and ovaries in cows suffering PUD compared to matched healthy cows (data records from the fourth to seventh postpartum weeks).

Group of cows	*n*	Uterine horns		Ovaries	
		Left	Right	Left	Right
Met	10	37.5 ± 10.6	43.75 ± 10.3	n.d.	12.5 ± 7.2
Healthy matched to Met	10	n.d.	n.d.	15.0 ± 15	20.8 ±
CE	17	9.4 ± 5.5	7.4 ± 4.0	9.4 ± 4.5	21.2 ± 6.2
Healthy matched to CE	17	n.d.	16.7 ± 11.0	16.1 ± 6.2	12.5 ± 3.5

n.d.: not done because no blood flow was detected. Values are expressed as mean ± standard error (*P* > 0.05, one-way ANOVA). Met, metritis. CE, clinical endometritis.

## Data Availability

The authors declare that the database in Excel format is available to interested veterinary and scientific personnel upon request by e-mail. In addition, all the data from the study are consolidated in tables and figures presented in this article. If you have any questions, please contact Juan G. Maldonado-Estrada e-mail: juan.maldonado@udea.edu.co.

## Supplementary Materials

Supplementary Materials Supplementary Figure 1: cross section diameter of uterine horns. (A and B) Left and right uterine horn diameter of healthy cows that have not developed PUD from the fourth to the tenth postpartum week. (C and D) Left and right uterine horn diameter between cows that developed PUD and healthy matched cows. Values are expressed in mm as mean ± SEM. There were no statistically significant differences between postpartum weeks in healthy cows or PUD versus healthy cows (*P* > 0.05). Supplementary Figure 2: Doppler spectral assessment of the right uterine artery. (A and B) RI and PI of healthy cows that did not develop PUD from the first to the tenth postpartum week. (C and D) RI and PI between cows that developed PUD and their corresponding healthy matched cows. Values are expressed as mean ± SEM. There were no statistically significant differences between postpartum weeks in healthy cows or PUD versus healthy cows (*P* > 0.05). Supplementary Figure 3: blood flow assessment of the right uterine artery. (A and B) TMAX and TMEAN values of healthy cows that did not develop PUD from the first to the tenth postpartum week. (C and D) TAMAX and TMEAN values between cows that developed PUD and their corresponding healthy matched cows. Values are expressed as mean ± SEM. There were no statistically significant differences between postpartum weeks in healthy cows or PUD versus healthy cows (*P* > 0.05). Supplementary Figure 4: D/S assessment of the right uterine artery. (A) D/S values of healthy cows that did not develop PUD from the first to the tenth postpartum week. (B) D/S values between cows that developed PUD and their corresponding healthy matched cows. Values are expressed as mean ± SEM. There were no statistically significant differences between postpartum weeks in healthy cows or PUD versus healthy cows (*P* > 0.05). Supplementary Figure 5: resistance index (RI) values in healthy cows according to the predominant structure in the ovaries. None, no follicles nor corpus luteum. Follicles, only the presence of follicles from 3.8 to 22 mm. Corpus luteum, presence of a corpus luteum. Ovarian structures were diagnosed by B-mode ultrasound. Values are expressed as mean ± SEM. RI was statistically higher in cows with a corpus luteum than those without structures or follicles (*P* < 0.05).
