# Supplementary material for: Ultrasonographic Screening of Dairy Cows with Normal Uterine Involution or Developing Postpartum Uterine Disease Using B-Mode, Color, and Spectral Doppler
Source: Vet Med Int. 2023 Sep 15;2023:2597332. doi: 10.1155/2023/2597332 (PMC10516695; doi:10.1155/2023/2597332)
Supplement: Supplementary Materials — Supplementary Figure 1: cross section diameter of uterine horns. (A and B) Left and right uterine horn diameter of healthy cows that have not developed PUD from the fourth to the tenth postpartum week. (C and D) Left and right uterine horn diameter between cows that developed PUD and healthy matched cows. Values are expressed in mm as mean ± SEM. There were no statistically significant differences between postpartum weeks in healthy cows or PUD versus healthy cows (P > 0.05). Supplementary Figure 2: Doppler spectral assessment of the right uterine artery. (A and B) RI and PI of healthy cows that did not develop PUD from the first to the tenth postpartum week. (C and D) RI and PI between cows that developed PUD and their corresponding healthy matched cows. Values are expressed as mean ± SEM. There were no statistically significant differences between postpartum weeks in healthy cows or PUD versus healthy cows (P > 0.05). Supplementary Figure 3: blood flow assessment of the right uterine artery. (A and B) TMAX and TMEAN values of healthy cows that did not develop PUD from the first to the tenth postpartum week. (C and D) TAMAX and TMEAN values between cows that developed PUD and their corresponding healthy matched cows. Values are expressed as mean ± SEM. There were no statistically significant differences between postpartum weeks in healthy cows or PUD versus healthy cows (P > 0.05). Supplementary Figure 4: D/S assessment of the right uterine artery. (A) D/S values of healthy cows that did not develop PUD from the first to the tenth postpartum week. (B) D/S values between cows that developed PUD and their corresponding healthy matched cows. Values are expressed as mean ± SEM. There were no statistically significant differences between postpartum weeks in healthy cows or PUD versus healthy cows (P > 0.05). Supplementary Figure 5: resistance index (RI) values in healthy cows according to the predominant structure in the ovaries. None, no follicles nor corpus luteum. Fo [file 2597332.f1.zip › Supplement Figure 4. Henao-Et Al-D-S values PUD and Healthy cows Nov08-2022.pptx]

## Slide 1
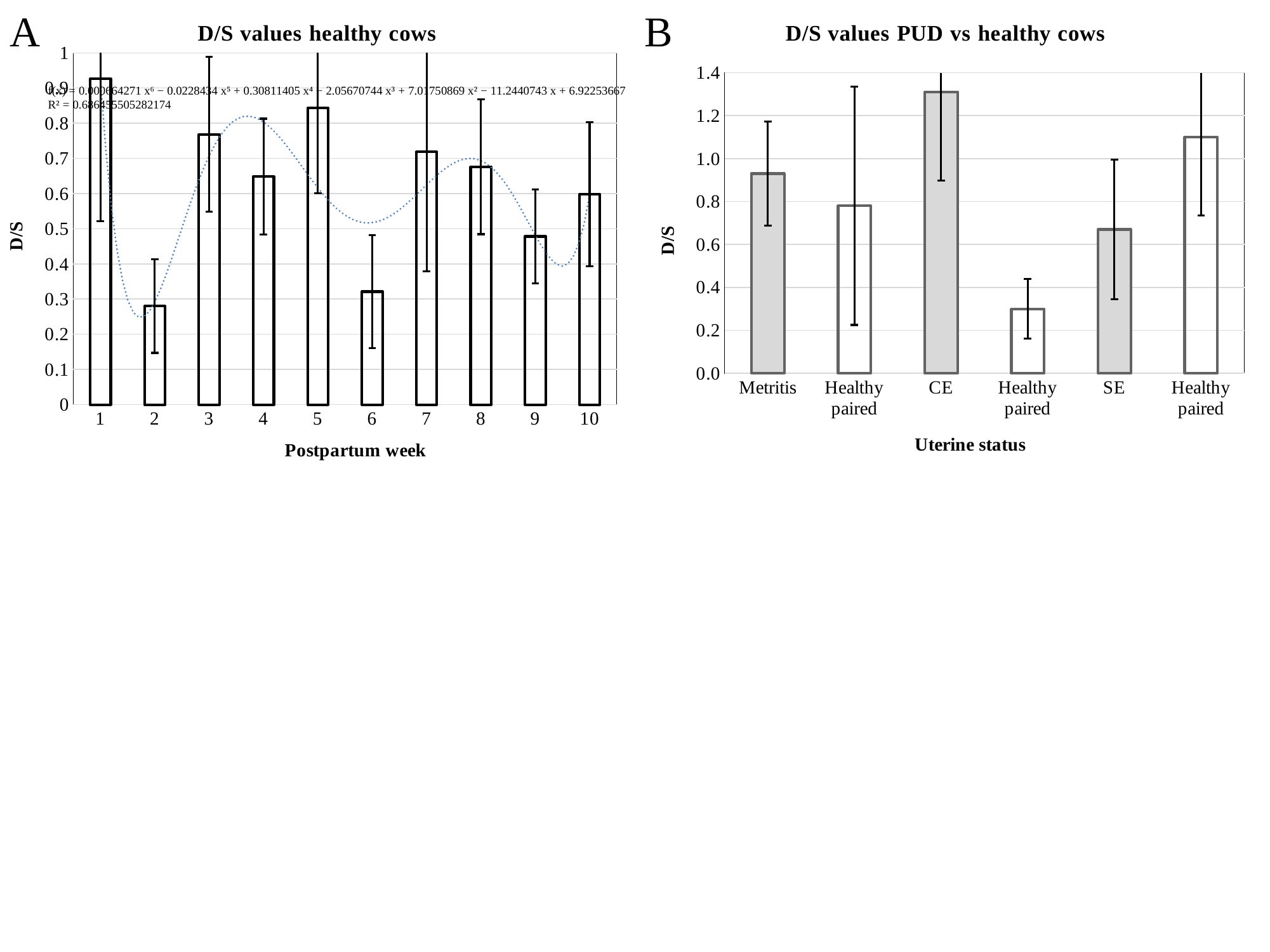

### Chart: D/S values healthy cows
| Category | D/S |
|---|---|A
B
### Chart: D/S values PUD vs healthy cows
| Category | D/S |
|---|---|
| Metritis | 0.93 |
| Healthy paired | 0.78 |
| CE | 1.31 |
| Healthy paired | 0.3 |
| SE | 0.67 |
| Healthy paired | 1.1 |A
